# Smart Modulators Based on Electric Field-Triggering of Surface Plasmon–Polariton for Active Plasmonics

**DOI:** 10.3390/nano12193366

**Published:** 2022-09-27

**Authors:** Jan Švanda, Yevgeniya Kalachyova, David Mareš, Jakub Siegel, Petr Slepička, Zdeňka Kolská, Petr Macháč, Štefan Michna, Václav Švorčík, Oleksiy Lyutakov

**Affiliations:** 1 Department of Solid State Engineering, University of Chemistry and Technology Prague, 166 28 Prague, Czech Republic; 2 Baumit, Spol. s.r.o., 250 01 Brandys nad Labem-Stara Boleslav, Czech Republic; 3 Research School of Chemistry and Applied Biomedical Sciences, Tomsk Polytechnic University, 634049 Tomsk, Russia; 4 Department of Microelectronics, Faculty of Electrical Engineering, Czech Technical University, 166 27 Prague, Czech Republic; 5 Faculty of Science, J. E. Purkyně University, 400 96 Usti nad Labem, Czech Republic; 6 Faculty of Mechanical Engineering, J. E. Purkyně University, 400 96 Usti nad Labem, Czech Republic

**Keywords:** smart materials, LIPSS, polymer, nanostructures, thin layers, plasmon excitation, sensor, modification, SERS

## Abstract

Design and properties of a plasmonic modulator in situ tunable by electric field are presented. Our design comprises the creation of periodic surface pattern on the surface of an elastic polymer supported by a piezo–substrate by excimer laser irradiation and subsequent selective coverage by silver by tilted angle vacuum evaporation. The structure creation was confirmed by AFM and FIB-SEM techniques. An external electric field is used for fine control of the polymer pattern amplitude, which tends to decrease with increasing voltage. As a result, surface plasmon–polariton excitation is quenched, leading to the less pronounced structure of plasmon response. This quenching was checked using UV–Vis spectroscopy and SERS measurements, and confirmed by numerical simulation. All methods prove the proposed functionality of the structures enabling the creation smart plasmonic materials for a very broad range of advanced optical applications.

## 1. Introduction

Noble metal nanostructures are revolutionizing modern science and gradually becoming more and more involved in numerous applications, ranging from medicine to optics [1,2]. Even in the photonics field, the unprecedented control of light at nanometer resolution can be considered as one of defining events of the last few decades [3,4]. The optical properties of noble metal nanostructures are defined by surface plasmon resonance and can be efficiently tuned by chosen materials, their shape or surrounding dielectric matrix [5,6,7]. Significant progress in the creation of nanostructures with desired optical response has been made during recent decades, but the gradual development of plasmonics provokes a new question—how to manipulate their optical response in situ? Indeed, quick and strong manipulation of plasmon resonance strength and position can open the way for the creation of high-capacity data storage, extremely quick light switching, or tunable and ultrasensitive detectors and sensors [8,9,10,11,12,13,14].

In this direction, most methods focus on effective changing of the medium surrounding the noble metal. The dielectric environment of noble metal nanostructures can be tuned via chemical (such as pH or ion strength) or physical (such as temperature, light illumination, or electric field) methods [15,16,17,18,19]. However, despite the many advantages, the complexity of their application together with relaxation time of the structures limits the practical utilization of these methods [20]. An alternative proposal of triggering of the metal intrinsic properties, by, e.g., changing the concentration of free electrons, was limited by the fact that the available range of optical modulation was significantly lower [21]. Taking into account the fact that the position of plasmon resonance is strongly affected by the interparticle distance, several methods were focused on tuning of the metal nanostructure arrangement [22,23]. As stated above, the plasmon–assisted optical switching can be reached through two approaches: tuning of plasmon–active nanoparticle environment or “re-arrangement” of plasmon-active nanostructure arrays. In the first case, good reversibility can be reached, but this approach does not allow high speed of modulation. In the second case, the common mechanical-based route is applied. The tuning was achieved either by the incorporation of metal nanostructures in the externally collapsed/swollen matrix or by their deposition on the stretchable surface with subsequent application of mechanical stress [24,25,26,27]. Most of the abovementioned works utilize mechanical strength, which limits the potential application of the prepared structures. The pattern parameters and dimension may also play an important role in further metallization processes [28,29,30]. The different types of nanopatterns, also with silver coating, were used in semiconductor junctions [31]. The shape of noble metal nanostructures is well known as the parameter which can affect the plasmonic resonance in a very strong manner [32]. Thus, it would be extremely interesting to use the tuning of the nanostructures’ shape for on-line modulation of their optical response [33,34]. There is a trend to substitute simple mechanical treatment by, for example, acoustic wave or electrostatic field [35,36].

In our previous works, we have investigated the electric field as an external stimulus [37] enabling efficient control of the substrate supporting the plasmonic nanostructures [38] and precise modulation of the distance and shape of gold nanoclusters. In this work, this technique is applied for creation of the surface plasmon–polariton supported pattern.

## 2. Experimental Methods

### 2.1. Materials

Lithium niobate (LiNbO_3_) substrates (z-cut) were supplied from Biotain Crystal Co. (Hong Kong, China) Polystyrene-*b*-polybutadiene-*b*-polystyrene (PS-b-PB-b-PS, *M*_w_ = 140,000 g mol^−1^, styrene 30 wt.%), and Rhodamine 6G (R6G) were supplied by Sigma-Aldrich (St. Louis, MO, USA). The deposition of Ag was accomplished using a Ag target (purity 99.99%, provided by Safina a.s, Vestec, Czech Republic).

### 2.2. Sample Preparation

Thin polymer films were prepared on the LiNbO_3_ substrates using a spin-coating procedure from 5 wt.% solution in toluene. The prepared samples were dried under ambient conditions for 24 h. The flat polymer surface was patterned by a KrF excimer laser (COMPexPro 50F, Coherent, Inc., Santa Clara, CA, USA, wavelength 248 nm, pulse duration 20−40 ns, repetition rate 10 Hz). The dried films were patterned by an excimer laser under the following parameters: angle from surface normal—50°, laser fluency—9 mJ·cm^−2^, number of laser pulses—3500. The pattern periodicity was 380 nm, the pattern amplitude 35 nm. As a result, the periodic surface structures were created on the PS-b-PB-b-PS surface. Experimental conditions were optimized to maximize available regularity of the periodic pattern on the polymer surface.

Silver was then deposited onto the patterned surface by vacuum evaporation using the tilted angle technique. The Ag evaporation was performed under 10^−4^ Pa with a deposition rate of 3 Ås^−1^. For the spatially selective Ag deposition, the samples were first oriented at an angle of 50° in relation to normal incidence of the evaporated silver atoms. Then, the Ag deposition was repeated after rotating the samples at 90° relative to the initial Ag deposition axis. The resulting Ag thickness was controlled by gravimetry. For SERS measurement, the typical Raman analyte–Rhodamine 6G (R6G)–was deposited from a methanol solution (10^−7^ M) using the spin-coating procedure at 1000 rpm for 10 s.

**Figure 1:**
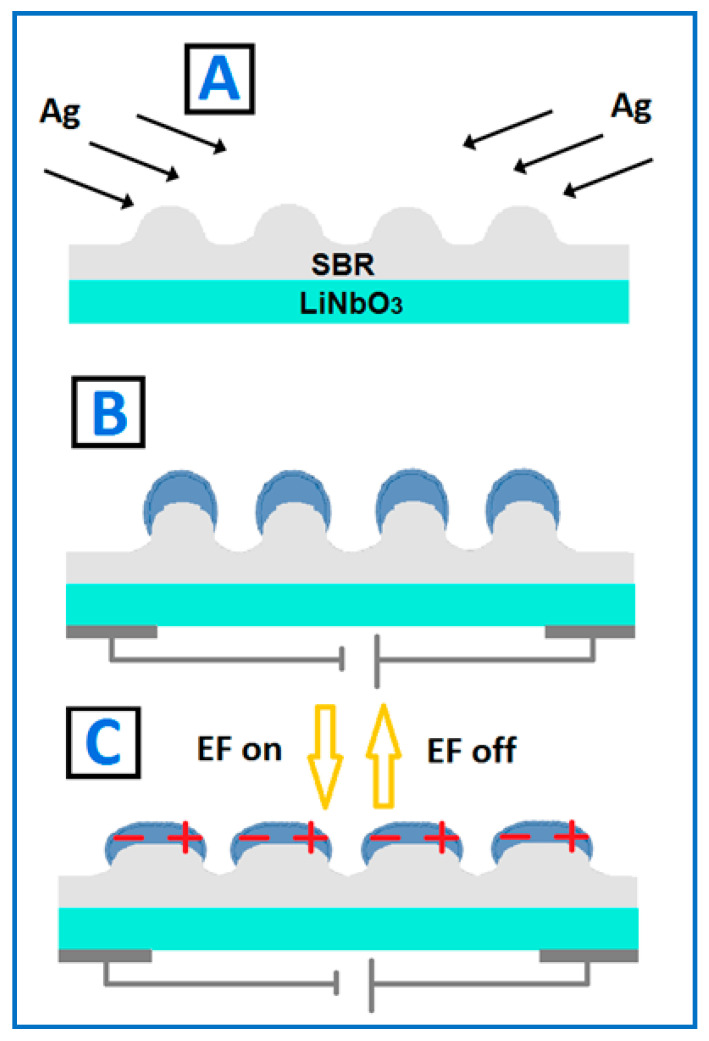
The schematic description of the sample preparation procedure and their supposed behavior. The thin PS–b–PB–b–PS polymer film was deposited on the piezo-electric substrate and patterned using the excimer laser (**A**). The tilted angle vacuum evaporation of Ag was applied to prepare an ordered array of c-shaped Ag nanostructures (**B**). The application of external voltage leads to stretching of the substrate and deformation of the polymer pattern. As a result, the Ag shape is transformed as depicted in (**C**).

Figure 1 shows a chart of the sample preparation procedure and expected behavior of the samples. The thin PS–b–PB–b–PS polymer film was deposited on the piezo-electric substrate and patterned using the excimer laser (Figure 1A). At an ambient temperature, the PS–b–PB–b–PS is in the rubber (elastic) state, so it is able to undergo “large” deformation without rupture. In the next step, the tilted angle vacuum evaporation of Ag was applied to prepare an ordered array of c-shaped Ag nanostructures (Figure 1B). This array can efficiently support excitation and propagation of surface plasmon polaritons (SPPs). As was proved before, the application of external voltage leads to stretching of the substrate and deformation of the polymer pattern. As a result, the Ag shape will be transformed as depicted in Figure 1C. Since the efficiency of SPP excitation on the periodical metal surface is a function of the pattern amplitude, it can be expected that shape modulation of the Ag nanostructures will lead to quenching of SPP excitation.

### 2.3. Measurement Techniques

Surface morphology of the samples was studied using AFM (Icon-Bruker, Billerica, MA, USA), working in peak force mode with a Scan–Asyst probe. Conductive AFM measurements were performed with a 3 V bias voltage, applied between the sample surface (after Ag deposition) and the AFM probe (Pt/Pd covered probe was used in this case).

UV/Vis spectra were measured using a Lambda 25 UV/Vis spectrometer (Perkin-Elmer, Waltham, MA, USA) ranging from 250 to 1000 nm with and without external voltage triggering.

SERS measurements were performed using a ProRaman-L spectrometer (laser power 15 mW, excitation wavelength 780 nm) with and without external voltage triggering.

For electric field (EF) measurements, two metal contacts (contact wide 1 mm, length 10 mm, between contact distance 5 mm) were deposited on the bottom side of the sample, connected to a voltage source. We would like to point out that we are aware that if the electrode distance is, e.g., 5 mm, and the applied electrical field is more than ~1 V/um, then the corresponding voltage would be more than 5 kV, which is a rather high value for practical application, however, these values were used, e.g., in monitors operating on the principle of a cathode electron-tube, and they are still used in the traditional electrophoretic separation of biomolecules.

## 3. Results and Discussion

**Figure 2:**
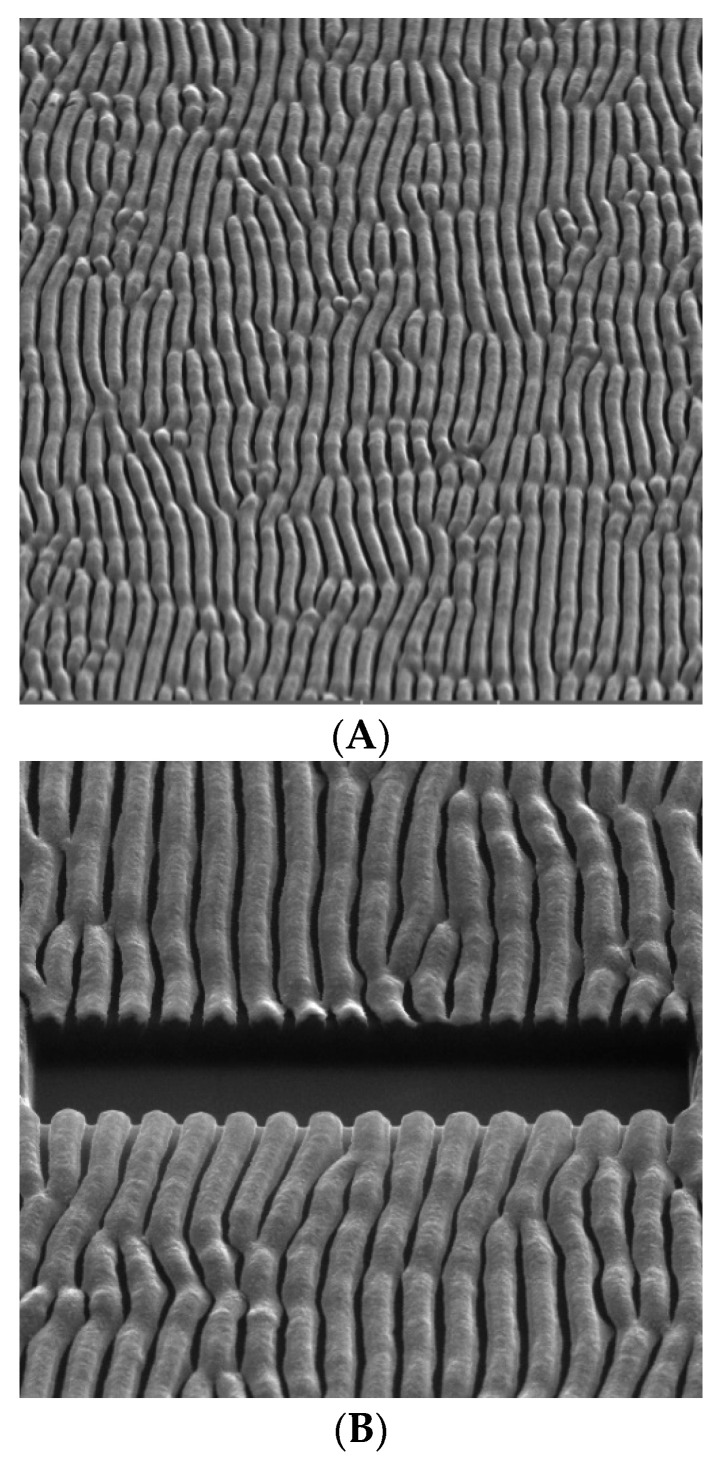
SEM image of Ag shadow evaporation on polymer (**A**) and FIB-SEM image of the same sample (**B**).

The presence of the ordered Ag c-shaped nanostructures was verified using the FIB-SEM technique. The Ag covered polymer pattern was cut and analyzed under the angle of 45° with respect to the surface plane. The FIB-SEM results are presented in Figure 2, which shows the SEM images taken of the pattern before (Figure 2A) and after the cutting (Figure 2B). The structure measured on the cut surface clearly displays the distribution of the Ag in the polymer pattern. More pronounced Ag brightness is due to its higher conductivity and roughness. It can be convincingly concluded that the array of the Ag c-shaped nanostructures was successfully created on the patterned polymer surface. The individual Ag nanostructures are well separated and their structure can efficiently support the excitation and propagation of SPPs.

**Figure 3:**
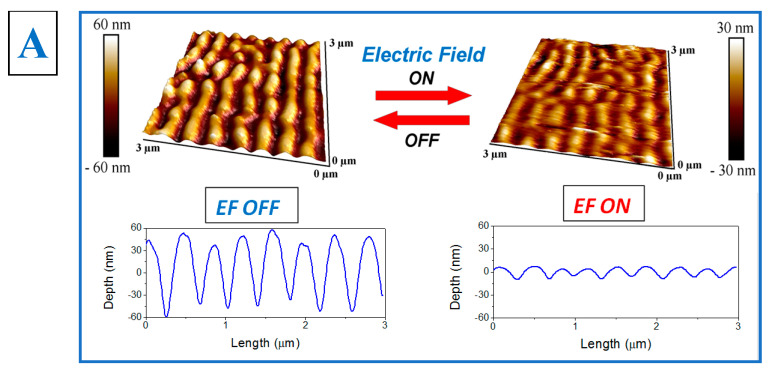

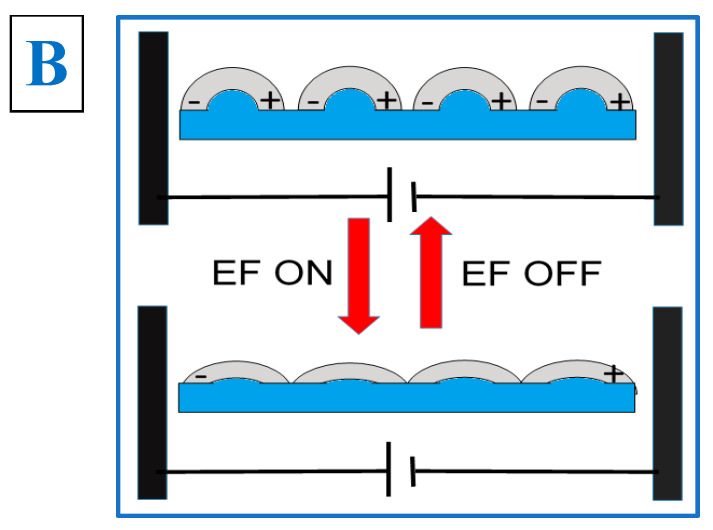
(**A**) AFM images (surface morphology) of Ag shadow evaporation on polymer taken with/without triggering by electric field (EF, in V/µm) and pattern amplitudes with/without EF. (**B**) Nanostructures’ polarization and appearance of out-of-plane electrostatic forces, inducing the structures’ flattening.

In the next step, the surface pattern was changed in order to modulate its interaction with the light. To do this, an electric field (EF) was applied via electrodes deposited at the sides of the substrate (see Figure 1). The changes in the polymer pattern morphology were tested using AFM morphology measurements, performed with and without the application of EF (Figure 3). The AFM measurements show that the application of EF leads to changes in the polymer pattern morphology (Figure 3A), which are more pronounced in comparison with expected ones. As is evident from Figure 3, the pattern amplitude decreases significantly. Since such large morphological changes cannot be explained by the substrate response only, we suppose that EF local intensity on the c-shaped edge induces charge redistribution in the nanostructure array, their polarization, and appearance of out-of-plane electrostatic forces, which may induce the structures’ flattening (Figure 3B).

**Figure 4:**
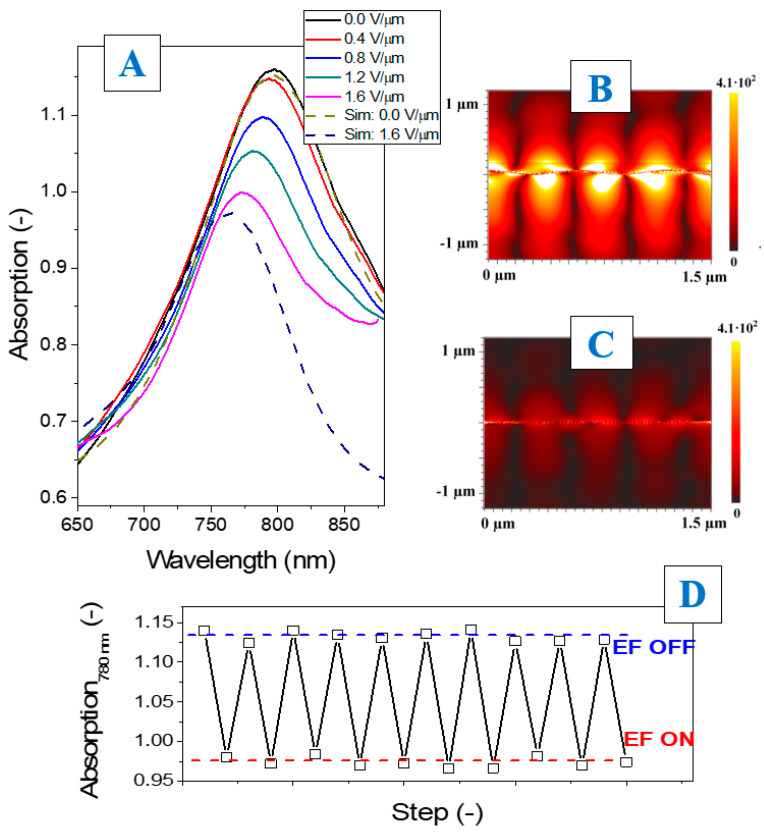
UV–Vis spectra and numerical simulation, performed on the c–shaped Ag arrays with/without the EF triggering (**A**); electric field distribution on the pristine (amplitude–50 nm) and EF triggered (amplitude–10 nm) Ag array (**B**,**C**); changes in absorption coefficient (at 780 nm) under subsequent EF switch on/off cycling (**D**).

Direct examination of SPP excitation was performed by UV–Vis spectroscopy and the results are compared with numerical simulations in Figure 4. In particular, Figure 4A shows the absorption spectra measured on the pristine array of the c-shaped Ag nanostructures and on the array triggered with EF of different intensities. The simulation (dashed lines) was performed for two critical cases—the pristine structure and the structure triggered with the maximal intensity of EF. The data for the simulation (i.e., the structures’ geometrical parameters) were extracted from the AFM measurements (Figure 3—cut-off profiles). As is evident from the simulation results, the appearance of a strong SPP-related absorption peak at 790 nm wavelength is expected. The measured results are in perfect agreement with the simulation–the absorption peak in the spectra of the pristine sample almost completely coincides with the simulation one. The application of external EF leads to a decrease in the pattern amplitude which was confirmed by the AFM measurements. At the same time, the absolute amplitude of the SPP peak decreases and it becomes “blue shifted” in excellent accord with simulation results. So, we can conclude that the SPP excitation is partially prohibited and tuning of the plasmonic structure response by EF was really reached.

Numerical simulation also allows us to present the efficiency of plasmon excitation as EF intensity near the plasmon surface (Figure 4B,C) for the pristine and maximally triggered samples. For this purpose, the 780 nm light wavelength was chosen for probing (taking into account further SERS experiments described below). Simulation results indicate that stronger EF focusing occurs in the case of the pristine sample. Decreasing of the pattern amplitude leads to apparent quenching of the plasmon excitation. Figure 4D shows the absorption behavior during several cycles of the structures’ triggering, where EF was repeatedly switched on/off. The absorption coefficient corresponding to the SPP maximum was chosen as a characteristic parameter in this case. As can be seen, during all triggering cycles, the absorption coefficient varies from 1.14 to 0.98 and the deviation between the cycles does not exceed the 5%, confirming in this way the excellent reproducibility of plasmon modulation. Moreover, the absorption coefficient returned to its pristine value in all cases, immediately after EF switching off regardless of the cycle number. This shows perfect stability of the structures’ triggering with the absence of any damage to the ordered Ag nanostructures in the process of plasmon modulation.

**Figure 5:**
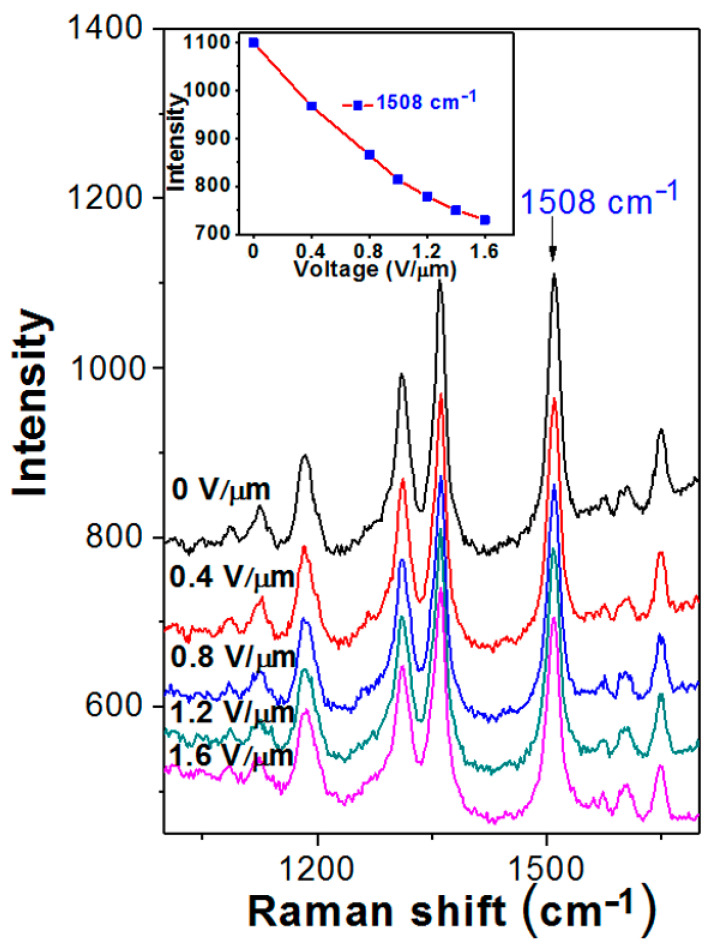
SERS study of plasmon activity by R6G response, measured on the array of c-shaped Ag nanostructures with/without the EF (V/µm) triggering.

Further examination of SPP triggering was performed using SERS spectroscopy. The intensity of the Raman response of targeted molecules (in our case, typical SERS marker R6G was used) is supposed to be proportional to the efficiency of plasmon generation. Therefore, the observation and valuation of the SERS response allows the estimation of the SPP excitation efficiency [39]. In Figure 5, the SERS spectra measured on the sample without triggering and under gradually increasing voltage are presented. As could be expected from the simulation and the results of UV–Vis spectroscopy, plasmon excitation is partially quenched due to decreasing of pattern amplitude when EF intensity increases. As a result, SERS intensity also decreases, as can be seen from Figure 5.

In particular, the intensity of the more pronounced SERS band, located at 1508 cm^−1^, decreases by 35%. On the other hand, the absorption coefficient (see Figure 4) measured at 780 nm decreases by ca 20%. Such a discrepancy could be explained by a contribution to the SERS response from the non-linear optical phenomena and local inhomogeneities on the almost flat Ag layer. In general, the SERS examination confirms quenching of SPP excitation, which is in good agreement with the previous results and expectations.

## 4. Conclusions

In this paper, the design and functionality of the on-line and electric field tunable plasmonic modulator are presented. The modulator consists of the ordered array of the c–shaped Ag nanostructures deposited on the highly flexible polymer layer (PS–b–PB–b–PS). The ordered array of the Ag nanostructures was prepared on a large-scale periodical structure created on the surface of PS–b–PB–b–PS using excimer laser light, followed by tilted angle Ag vacuum evaporation. Such a structure can efficiently support the excitation and propagation of surface plasmon–polariton, visible as a strong absorption peak, located near 800 nm. When the external EF was applied to the system, the deformation of the Ag covered pattern was observed, due to EF-induced synergy of polarization of the c-shaped Ag structure, interaction of created dipoles with external EF, their mutual interaction, and further support by the piezo–response of neighboring substrate. As a result, the pattern amplitude significantly decreases, leading to the strong change in the SPP excitation phenomenon. In particular, it was found that a slight “blue shift” and appreciable quenching of the SPP–related absorption peak occurs. Additionally, finite difference time-domain numerical simulation, performed for the pristine and electrically triggered array of c-shaped silver nanostructures, confirms the SPP quenching. Finally, plasmon triggering was confirmed by the SERS measurements too.

## Data Availability

Data is contained within the article.
